# Gender-Based Disparities in Knowledge, Attitudes, and Practices Among Type-II Diabetes Patients in Punjab, India: Insights for Tailored Interventions

**DOI:** 10.2174/0113816128409456251008231638

**Published:** 2025-10-24

**Authors:** Sandeep Kaur, Sidharth Mehan, Ghanshyam Das Gupta

**Affiliations:** 1 Department of Pharmaceutics, ISF College of Pharmacy, Moga, Punjab, India;; 2 Research Scholar, IK Gujral Punjab Technical University, Jalandhar, Punjab, India;; 3 Department of Pharmacology, ISF College of Pharmacy, Moga, Punjab, India (Affiliated to IK Gujral Punjab Technical University, Jalandhar, Punjab, 144603, India)

**Keywords:** Type II diabetes mellitus, gender disparities, knowledge-attitude-practice, self-management, healthcare access, diabetes management

## Abstract

**Introduction:**

Managing Type II Diabetes Mellitus (T2DM) can be extremely difficult, especially in diverse populations where patient outcomes may be impacted by gender differences. Understanding patients' knowledge, attitudes, and practices (KAP) is essential for creating focused interventions. This study aims to evaluate the KAP of T2DM patients attending outpatient clinics in Moga, Punjab, India, with a focus on gender disparities.

**Methods:**

A cross-sectional study was conducted among 500 T2DM patients (197 females and 303 males). Inclusion criteria were T2DM patients aged above 18 years, while pregnant and breastfeeding women were excluded. Data were analyzed using SPSS version 25, applying the Mann-Whitney U, Kruskal-Wallis, Chi-square, and Spearman’s correlation tests.

**Results:**

Poor knowledge and attitude scores were observed in 32.8% and 37.4% of patients, respectively, while 51.7% displayed fair practice scores. A gender-wise analysis revealed that males had higher proportions of good knowledge (33.7%) and attitude scores (50.2%) compared to females (20.8% and 36.0%, respectively). In terms of practice, both genders reported similar poor scores (31%).

**Discussion:**

Males had significantly higher knowledge and attitude scores, with better awareness of T2DM risk factors, complications, and management strategies, likely due to higher educational attainment and greater access to healthcare resources, but both genders faced challenges in translating knowledge into self-care practices. Correlation analysis revealed a positive association between KAP scores and clinical parameters.

**Conclusion:**

The study underscores the need for tailored educational programs that incorporate socio-cultural considerations and improved access to healthcare resources, which are crucial for bridging gender gaps in diabetes self-management.

## 1 INTRODUCTION

Type II Diabetes Mellitus (T2DM) remains a major global health challenge, with its prevalence steadily rising over the past few decades [1-3]. In 2021, the prevalence of diabetes among individuals aged 20 to 79 years was 10.5%, affecting approximately 536.6 million people worldwide. This number is expected to increase to 12.2% by 2045, impacting a total of 783.2 million individuals [4-6]. The highest relative increase in diabetes prevalence from 2021 to 2045 is expected in middle-income countries, with an estimated growth rate of 21.1%. Global healthcare costs related to diabetes were around 966 billion USD in 2021 and are projected to reach 1,054 billion USD by 2045 [7-10]. A large cross-sectional study conducted by the Indian Council of Medical Research (ICMR-INDIAB) between 2008 and 2020 reported a national diabetes prevalence of 11.4% and a prediabetes prevalence of 15.3%. In Punjab, a study revealed that approximately one in ten adults has diabetes, with a prevalence rate ranging from 8.7% to 11.2% [11-14]. These findings highlight that Punjab has a higher burden of diabetes and emphasize the need for more specific interventions and preventive strategies.

Complications associated with diabetes increase mortality and morbidity rates among the diabetic population [15, 16]. The complex nature of Type II Diabetes Mellitus (T2DM) requires a thorough understanding of its risk factors, management, and related parameters to develop effective prevention and control strategies [17-19]. The management of T2DM necessitates a combined approach involving lifestyle modifications and pharmaceutical interventions [20-22]. Furthermore, demographic factors can affect a person's knowledge, attitude, and practice (KAP) related to diabetes, which in turn influence their overall health outcomes.

Recent studies focusing on evaluating the KAP of patients based on gender have shown the critical need for gender-based awareness programs aimed at disease prevention, diagnosis, mitigation of risk factors, and reduction of complications associated with the condition. Both males and females face different barriers to lifestyle modification according to their condition, which may contribute to varying levels of knowledge, attitude, and practice toward the disease [23-25]. Furthermore, it has been suggested that individuals with T2DM who have a good understanding of their condition are more likely to achieve better blood glucose control [26-29]. Additionally, unfavorable attitudes have been associated with poor blood glucose control and a higher risk of complications [30-34].

KAP studies are essential for understanding the factors that influence diabetes management. They can help identify vulnerable populations at higher risk of developing Type II Diabetes Mellitus (T2DM) and its complications, thus enabling targeted preventive measures [35-38]. Furthermore, understanding the links between demographic factors and KAP can guide the development of patient-centered interventions and educational programs aimed at enhancing self-management [39-41]. Gender, in particular, plays a crucial role, as male and female patients often differ in health literacy, awareness of risk factors, and adherence to lifestyle modifications [42-44]. For example, studies indicate that women may face unique barriers to accessing healthcare and achieving optimal diabetes management, partly due to socioeconomic and cultural factors that limit their ability to adopt recommended lifestyle changes. Similarly, men often demonstrate greater awareness of lifestyle risk factors and preventive measures, which can result in differences in health outcomes [45-49].

This study seeks to address this gap by assessing the knowledge, attitude, and practice metrics of patients with Type II Diabetes Mellitus (T2DM) through a gender-sensitive lens, acknowledging the need for tailored interventions to meet the distinct needs of male and female patients. This study also explores the relationship between demographic factors such as age, education level, marital status, health insurance coverage, occupation, and frequency of clinic visits, and KAP scores, offering insights to inform more tailored and effective diabetes management approaches.

Recent studies in Indian clinics have reported a high incidence of microvascular complications in patients with uncontrolled Type II Diabetes Mellitus (T2DM). In a community-based study in Kerala involving 3,000 patients, the prevalence of diabetic retinopathy was found to be 28.9% and peripheral neuropathy 48.5%, both of which were closely linked to higher HbA1c levels and longer duration of diabetes [50].

These findings were also confirmed by the nationwide SMART-India multicenter screening, which estimated the national prevalence of diabetic retinopathy to be 12.5%. This prevalence was significantly higher in individuals with clinically diagnosed diabetes (15.5%) compared with those with undiagnosed diabetes (8.0%); 4.0% of respondents had vision-threatening diabetic retinopathy (VTDR). Overall, these data highlight the urgency of establishing large-scale screening programs to identify microvascular complications in the Indian population with T2DM and to manage these complications [51].

This cross-sectional analysis aims to identify specific areas where healthcare providers can improve educational and motivational strategies to promote better self-management among T2DM patients, ultimately reducing complications and enhancing the quality of life across genders.

## 2 MATERIALS AND METHODS

### 2.1 Study Setting and Design

This cross-sectional study was conducted among Type II Diabetes Mellitus (T2DM) patients visiting different healthcare facilities in Punjab, North India. The research was carried out between February 2023 and July 2024.

#### 2.1.1 Study Population

**Fig. (1):**
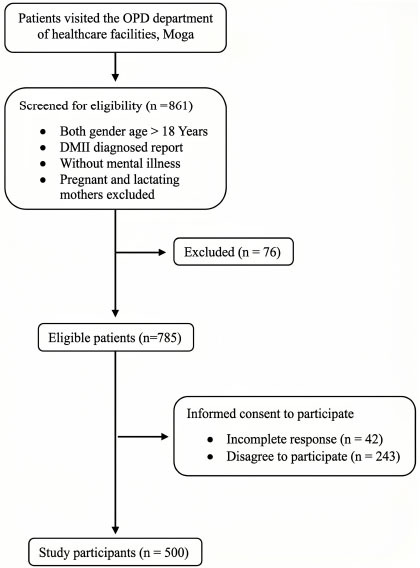
The figure presents a study framework, including screening procedures, eligibility criteria, and sampling methodology. A cross-sectional study with non-probability purposive sampling methodology was used. Pilot testing, reliability, internal consistency, and face validity of study tool.

The current study recruited T2DM patients attending primary healthcare facilities in the Moga district of Punjab, India. A total of 500 participants were selected for the study using non-probability purposive sampling, following the specified inclusion and exclusion criteria (Fig. **1**). All patients were thoroughly briefed about the study before participation, and informed consent was obtained.

### 2.2 Inclusion and Exclusion Criteria

The inclusion criteria included all patients above 18 years of age with a clinical diagnosis of Type II Diabetes Mellitus (T2DM) and a medical record of HbA1c (glycated hemoglobin), FSL (fasting sugar level), and RBS (random blood sugar). Patients with mental illness and those who did not provide informed consent were excluded. Pregnant or lactating women were also excluded.

Fig. (**1**). Overview of study participant selection and sampling procedure.

The study tool was pretested with 25 participants to ensure that the questions were clear and easily understandable. The test-retest method was used to assess the consistency of scores over a short period. Participants completed the survey twice, with a one-week interval between sessions. Pearson's correlation analysis compared the scores from both sessions, revealing strong stability (knowledge: 0.992, attitude: 0.967, practice: 0.885; *p <* 0.001). The reliability, measured by Cronbach's alpha, indicated good internal consistency (knowledge: 0.938, attitude: 0.793, and practice: 0.880).

### 2.3 Data Collection and Data Analysis

For data collection, the study used a validated questionnaire on knowledge, attitude, and practice regarding T2DM [52]. The questionnaire consisted of two parts (see Supplementary Material). The first part focused on the informed consent form, while the second part covered the demographic profile of the respondents and KAP variables. Knowledge of patients about their disease was evaluated with 14 multiple-choice questions covering disease knowledge, risk factors, treatment, and complications. One point was awarded for each correct answer and zero for each incorrect answer. The total scores were then calculated out of 40 points. The score range for knowledge was categorized as 0-12 (poor), 13-26 (fair), and 27-40 (good). Responses to the items assessing patients’ attitudes toward the illness included a total of nine questions. Attitude was assessed using a five-point Likert scale, with responses ranging from 1 (strongly disagree) to 5 (strongly agree), with intermediate values for the other options. The highest possible score was 45, and the score range was classified as less than 15 (poor), 15-30 (fair), and 31-45 (good). In the final section, five categorical questions were included, with response options of 0 (not at all), 1 (yes, occasionally), and 2 (yes, frequently). These questions assessed the respondents' practices toward diabetes. The maximum possible score was 7, and the score range was categorized as 0-2 (poor), 3-5 (fair), and 6-7 (good). The questions were administered face to face, and illiterate patients were given verbal assistance, with their answers recorded on the questionnaire.

Data analysis was conducted using the Statistical Package for the Social Sciences (SPSS) version 25 for Windows, with a 95% confidence interval (CI). The normality of data distribution was assessed using the Kolmogorov-Smirnov and Shapiro-Wilk tests. As determined by these tests, continuous variables (KAP scores) were not normally distributed and were expressed as median (Q2) ± interquartile range (IQR: Q1-Q3), while categorical variables were presented as frequencies and percentages. Differences in continuous variables between more than two groups were examined using the Kruskal-Wallis test, while the Mann-Whitney U test was used to test significant differences between two groups. The Chi-square test was applied to compare categorical variables (KAP scores) among males and females, which were expressed as frequencies and percentages, while Spearman’s rank correlation coefficient was used to analyze relationships between KAP variables and demographic variables.

### 2.4 Ethical Approval and Consent to Participate

The study-related activities were initiated after obtaining approval from the Institutional Ethics Committee (IEC) of the ISF College of Pharmacy, Moga (ECR/296/Indt/PB/2023/ISFCP/39), in accordance with the ethical principles outlined in the Declaration of Helsinki. The study details were clearly communicated to the participants, and informed consent was obtained from all individuals. Participation did not affect their treatment process. Patient information was kept confidential and stored securely. The SAGER Guidelines were followed.

## 3 RESULTS

### 3.1 Patient’s Demographics

The demographic profile of the recruited patients is summarized in Table **1**. A total of 500 patients with Type II diabetes, aged 31-90 years, and weighing between 41 and over 101 kg, were evaluated. The study included 197 female and 303 male patients. Regarding education, among the 500 participants, 17% had completed graduation, while 5.8% held a master’s degree. Additionally, most of the female participants in the study were illiterate, as shown in Fig. (**2**).

Furthermore, most of the recruited patients were employed (44%), while 22% were unemployed, 21.2% were housewives, and 10.4% were retired. Additionally, 2.4% had other occupations, and their gender distribution is shown in Fig. (**3**). Among the recruited patients, 254 had health insurance, whereas 246 did not, with the majority being female, as illustrated in Fig. (**4**). Most of the recruited patients (81.4%) had HbA1c blood levels above 6.5%.

Among the 500 recruited patients, 164 (32.8%) had poor knowledge scores (<13), 193 (38.6%) had fair knowledge scores (13-26), whereas 143 (28.6%) had good knowledge scores (27-40). Weight, age, marital status, gender, occupation, education, family history, health insurance, physical activity, and frequency of clinic visits were significantly associated (*p <* 0.05) with knowledge scores (Table **1**). Patients aged 31-40 reported the highest knowledge score (median score: 23). Furthermore, males showed higher knowledge scores compared to females. Married and unmarried patients reported higher knowledge scores than divorced and widowed patients. Patients with bachelor’s and master’s degrees showed significantly higher knowledge scores than those with other educational levels. Furthermore, higher knowledge scores were reported among retired patients, those who had health insurance, a family history of diabetes, visited the clinic every three months, and had high levels of physical activity, as shown in Table **1**. The detailed responses regarding patients’ knowledge, stratified by gender, are presented in Table **2**. More male patients were able to identify smoking (*p <* 0.001) and alcohol (*p =* 0.003) as lifestyle risk factors for T2DM compared to females. Similarly, more males recognized exercise (*p =* 0.029) as a management option for T2DM than females. Additionally, more males identified sexual dysfunction (*p =* 0.019), kidney failure (*p <* 0.001), neuropathy (numbness) (*p =* 0.017), and eye problems or blindness (*p =* 0.001) as complications of T2DM compared to females. However, more females were able to identify hypoglycemia (low blood sugar levels) associated with dizziness, nausea, and tremors (*p <* 0.001) as complications of T2DM compared to males. More males identified weight reduction (*p =* 0.004) as a lifestyle modification compared to females. KAP metrics by participants’ gender are shown in Fig. (**5**). The data indicate that male patients and those with higher education levels scored higher on the knowledge test, demonstrating a gender- and education-related difference in diabetes awareness.

Furthermore, regarding attitude, 187 patients (37.4%) had poor attitude scores (<15), 90 patients (18%) had fair attitude scores (15-30), and 223 patients (44.6%) had good attitude scores (31-45). The attitude score was significantly (*p <* 0.05) influenced by weight, age, marital status, education, gender, occupation, family history, health insurance, medical history, physical activity, and frequency of clinic visits. Attitude scores were higher among T2DM patients aged 31-40, males, married individuals, those with bachelor’s and master’s degrees, individuals with health insurance, patients with a family history of T2DM, recently diagnosed patients, and those with high levels of physical activity (Table **1**). Gender significantly influenced various attitude items; for example, when asked whether family members should be screened for T2DM, 67.1% of males strongly agreed compared to 32.9% of females (*p <* 0.0001) (Table **3**). These findings underscore the importance of demographic and socioeconomic factors in shaping patient attitudes toward diabetes, highlighting the need for personalized education and support strategies that address gender, education level, and healthcare access.

Poor practice scores (0-2) were reported by 155 patients (31%), fair practice scores (3-5) were recorded for 256 patients (51.2%), and good practice scores were observed in 89 patients (17.8%). Age, educational status, marital status, occupation, family history, health insurance, physical activity, and frequency of clinic visits significantly (*p <* 0.05) affected all practice items. Detailed practice responses of patients stratified by gender are presented in Table **4**. Gender did not have a significant effect on any of the practice items. Although there were substantial differences in knowledge and attitudes between different groups, self-care practices were relatively low among both genders, indicating a significant gap between self-care awareness and actual behavior.

The correlation analysis among various parameters revealed that knowledge scores were significantly correlated with FBS (fasting blood sugar) (*p =* 0.014), RBS (random blood sugar) (*p =* 0.007), age (*p <* 0.0001), attitude scores (*p <* 0.0001), and practice scores (*p <* 0.0001). Attitude scores were significantly associated with HbA1c (glycated hemoglobin) levels (*p =* 0.003) and age (*p <* 0.0001). Furthermore, we observed a significant correlation between practice scores, FBS (*p =* 0.017), and age (*p =* 0.003). Subsequently, all three knowledge scores, attitude scores, and practice scores were significantly (*p <* 0.0001) correlated with each other (Table **5**). A correlation analysis of the KAP data revealed a significant association between knowledge, attitude, and practice scores and several clinical and demographic variables. Knowledge scores were positively related to RBS values, age, attitude, and practice scores, indicating that the more patients know, the greater their awareness and the better their behavior. The correlation of attitude scores with HbA1c levels and age supports the suggestion that more desirable attitudes are associated with improved glycemic control and are more common among older individuals. On the other hand, practice scores showed strong correlations with FBS, age, and knowledge and attitude scores, suggesting that better self-care practices can be achieved among knowledgeable individuals who have positive beliefs about disease-related activities. Collectively, these findings support the hypothesis that reinforcing knowledge and attitude contributes to improved diabetes management behaviors.

## 4 DISCUSSION

This hospital-based cross-sectional study evaluated the KAP of 500 DMII patients in Moga, Punjab, India. The findings from this study revealed significant gender-based differences in the KAP metrics among DMII patients, with male patients demonstrating higher scores in knowledge and attitude toward DMII management.

The assessment of KAP scores in our study provides essential insights into the patients' understanding of diabetes, their attitudes toward self-care, and their actual lifestyle and self-management practices [53-56]. In our study, patients with degrees showed better knowledge scores than patients without degrees. The disparity in educational levels, with 32.2% of patients being illiterate and only 17% having completed graduation in the study population, could significantly influence the knowledge scores. Therefore, there is a need for improved patient education and counseling programs. Individuals with lower education levels may have limited access to diabetes-related information and may find it challenging to understand and effectively adopt necessary self-care practices. A recent study found that individuals with a college-level education or higher exhibited a significantly higher likelihood of possessing good knowledge regarding diabetes mellitus. The analysis revealed an adjusted odds ratio of 3.70 (95% confidence interval: 1.26 to 10.85), indicating a strong and positive link between higher educational qualifications and a better understanding of diabetes mellitus [57-60]. Addressing this educational gap should be a priority when designing interventions and educational programs to empower patients with the knowledge and skills needed for effective diabetes management. Women from lower socioeconomic backgrounds have less access to formal education, which can restrict their ability to understand and apply health information. Our data support this, showing that most female patients in this study were illiterate. In contrast, more male patients had attained higher educational qualifications, such as graduate and master’s degrees. Educational attainment has been positively associated with better health literacy, which may explain why men were more informed about risk factors, complications, and management strategies for DMII. Moreover, social and cultural norms can restrict women’s access to health resources, including information on diabetes management. In some cultures, health discussions or decisions are often led by male family members, which may limit women’s exposure to health education. This can create an imbalance in knowledge and self-care practices among female patients, leading to lower awareness of key aspects of diabetes management, such as the importance of regular exercise and lifestyle changes.

Previous literature indicates that female diabetes patients tend to have superior knowledge of DMII, show more positive attitudes, and adhere more consistently to eating healthier meals than males [61-64]. However, it has been reported that females with diabetes experience more significant constraints in their daily lives because of emotional and physical issues compared to men with diabetes [65-68]. Additionally, our data reveal a notable gender disparity, with a significantly higher percentage of males demonstrating better knowledge scores regarding preventive and curative actions for DMII. In contrast, females were more aware of complications associated with high blood glucose, such as dizziness and nausea. These differences may be due to varying levels of health literacy, educational access, and cultural factors that influence health knowledge between genders. This gender-based difference suggests that males, in this context, might be more proactive or more convinced about the importance of early screening for diabetes within their families. This finding raises questions about how effective current diabetes education and outreach efforts are, especially in reaching and engaging female patients. It suggests tailored interventions that address the specific informational needs and barriers faced by women with diabetes. Policymakers should create gender-sensitive educational materials and programs. These resources should cover not only the physiological aspects of diabetes but also incorporate socio-cultural factors that may influence women's health behaviors. Digital health technologies and telemedicine platforms can expand the reach of diabetes education and support services to female patients, particularly those facing geographical or logistical constraints [69-72]. Mobile applications, online forums, and virtual support groups can provide easy access to information, monitoring tools, and peer support, empowering women to manage their diabetes from the comfort of their homes.

In terms of attitude, male patients also demonstrated a more proactive stance toward diabetes management. For example, more males strongly agreed on the importance of screening family members for DMII, indicating a broader sense of responsibility in managing the condition. This finding might indicate a higher focus on health concerns among men or a greater capacity to seek support from family networks. Conversely, women could face additional responsibilities, such as caregiving roles, which may restrict their ability to prioritize their health needs, thereby affecting their attitudes and adherence to recommended health practices.

Our study also found that gender did not significantly affect practice scores, indicating that although men had higher knowledge and attitude scores, both male and female patients faced difficulties in translating knowledge into consistent health practices. Factors such as time constraints, family responsibilities, and limited access to healthcare facilities may contribute to this, especially for women who often juggle household duties and may have less autonomy in seeking healthcare. Our study's distribution of practice scores suggests that more than half of the patients had fair practice scores, indicating moderate adherence to self-care practices. However, the significant percentage of patients with poor practice scores highlights the need for interventions to improve self-management behaviors. Additionally, the high rate of patients with inadequate glycemic control (HbA1c > 6.5%) calls for a more robust healthcare infrastructure, including better access to diabetes management resources and regular monitoring. A study conducted in Bijapur, Karnataka, found that many participants recognized diabetes as a serious health issue and understood the important role that diet plays in managing it. This shared understanding indicates that the respondents not only saw diabetes as a serious condition but also valued the importance of dietary choices in controlling the disease effectively [73, 74]. This awareness among the respondents demonstrates the importance of informed and proactive self-management approaches to diabetes. A systematic review of randomized controlled trials on multi-component self-management interventions for type II diabetes showed positive effects on diabetes-specific quality of life (QoL), knowledge improvement, and behavior, particularly with collaborative learning approaches [75, 76]. Another recent systematic review examined how diabetes self-management support and education affect glycemic control in adults with type II diabetes. Its results revealed an average absolute decrease in HbA1c levels of 0.57, indicating a significant decline in glycemic levels [77-80].

The positive correlation between attitude scores and HbA1c levels emphasizes the importance of psychological factors in glycemic control, showing that individuals with more optimistic attitudes tend to have better blood sugar management, as demonstrated previously [81, 82]. The role of age in shaping attitudes may be linked to the influence of life experience and maturity on patients' perspectives and health-related behaviors. Additionally, the strong correlations between practice scores, FSL, and age highlight the significance of age and blood sugar levels in guiding patients' self-care practices. Older individuals tend to follow self-management practices more strictly [83, 84]. Lastly, our study's interconnectedness of knowledge, attitudes, and practices highlights the holistic nature of diabetes management.

## CONCLUSION

The study at hand is a gender-sensitive, systematic analysis of KAP among individuals with DMII in India, specifically in the province of Punjab. The Chi-square test revealed significant differences between male and female participants with regard to both knowledge and attitude scores, with males portraying statistically significantly higher diabetes-related awareness and positive management attitude scores. However, similar and suboptimal patterns of self-care were observed across the genders, showing a miscorrespondence between thinking and acting.

Unlike other studies that examine only overall KAP tendencies or disregard gender-based segregation, this study prioritizes the influence of socio-demographic factors such as education, employment, and insurance status on the outcomes of diabetes management. Analyzing through a gender-sensitive perspective, the analysis highlights the unique struggles among women who live in rural and semi-urban areas, a factor that can be used to inform the creation of demographically tailored interventions and public health policies that are culturally competent.

Taken together, the findings demonstrate the importance of multicomponent diabetes management systems that go beyond raising awareness and include behavioral interventions, access to care, and community involvement. Educational programs that integrate study programs, digital technology, and neighborhood relations are more likely to successfully transfer the acquired knowledge into continuous practice.

## STUDY LIMITATIONS

There are various limitations to the present study. To begin with, it can only be examined among patients with DMII, which further limits the application of the findings to patients with Type I, gestational, or other forms of diabetes. Second, the data were collected through self-reported questionnaires, which are prone to recall and social desirability bias, especially in the reported attitudes and practices. Third, since the study used a cross-sectional design, no causal conclusions can be drawn. Fourth, the external validity of the results to other geographic contexts may be limited by cultural peculiarities specific to Punjab. Therefore, the authors suggest follow-up longitudinal and interventional studies to analyze the long-term effects of gender-sensitive educational and clinical programs.

## Figures and Tables

**Fig. (2):**
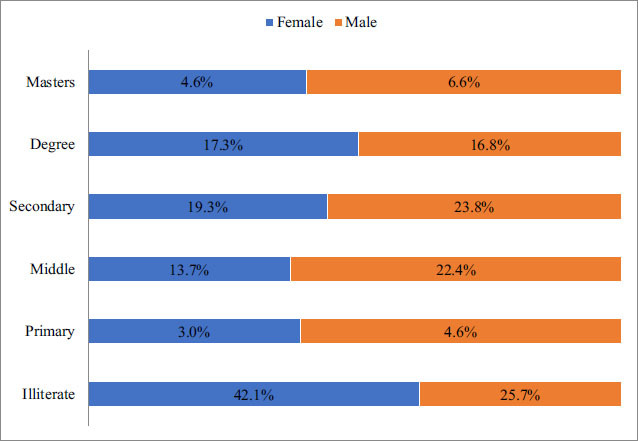
Educational demographics of participants based on gender in the clinical trial.

**Fig. (3):**
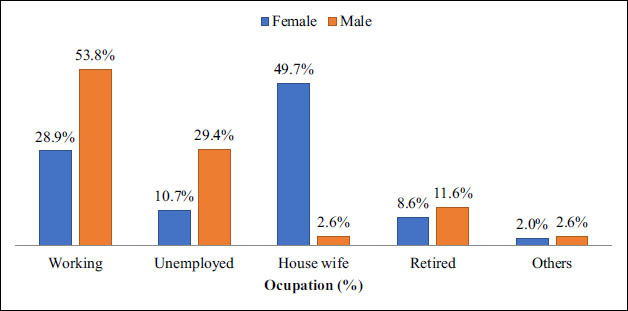
Occupation demographics of participants in the clinical trial.

**Fig. (4):**
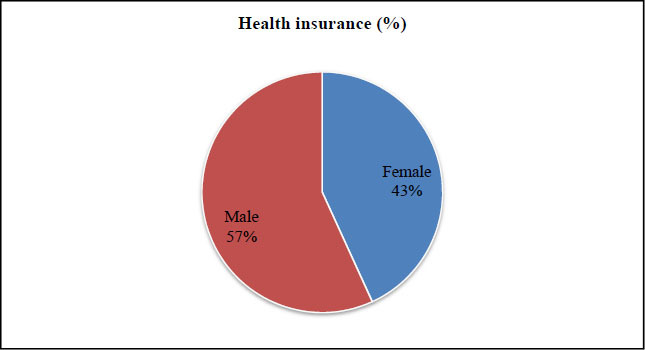
Health insurance status of participants in the clinical trial. Association of demographic parameters with knowledge, attitude, and practice.

**Fig. (5):**
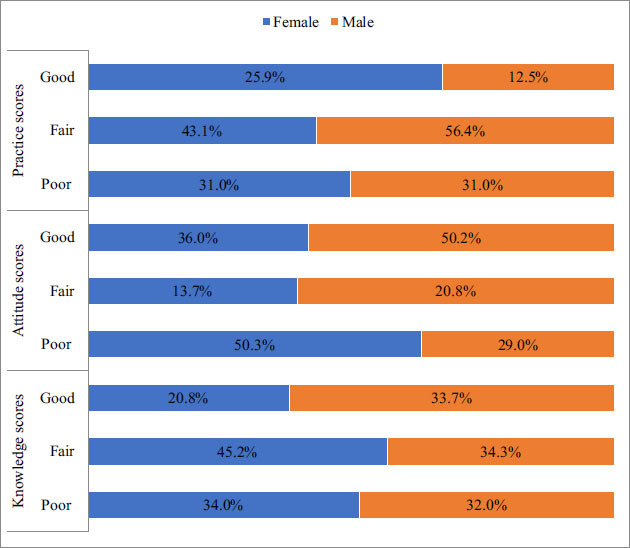
KAP scores of participants in the clinical trial.

**Table 1:** Descriptive demographic characteristics and KAP scores of type II diabetes patients participated in this study, (n = 500).

**Variables**		**n**	**%**	**Knowledge Scores**	***p*-value**	**Attitude Scores**	***p*-value**	**Practice Scores**	***p*-value**
				**Median (Q1, Q3)**		**Median (Q1, Q3)**		**Median (Q1, Q3)**	
Weight of the patient	41-50	16	3.2	15 (7, 23)	0.002	21 (14, 32)	0.039	2 (1, 5)	0.050
	51-60	50	10.0	17 (14, 28)		30 (14, 38)		4 (3, 5)	
	61-70	123	24.6	15 (9, 24)		28 (14, 32)		3 (2, 5)	
	71-80	176	35.2	18 (14, 29)		31 (15, 37)		4 (2, 5)	
	81-90	117	23.4	17 (11, 26)		29 (15, 37)		4 (2, 5)	
	91-100	16	3.2	20 (17, 28)		21 (14, 39)		4 (3, 6)	
	>101	2	0.4	7 (6, 8)		26 (24, 27)		2 (2, 2)	
Age group	31-40	18	3.6	23 (17, 34)	0.002	38 (15, 41)	0.001	2 (2, 2)	<0.001
	41-50	102	20.4	18 (10, 29)		32 (15, 38)		3 (2, 5)	
	51-60	170	34.0	18 (13, 28)		30 (15, 37)		4 (3, 5)	
	61-70	131	26.2	16 (9, 26)		28 (14, 36)		4 (3, 5)	
	71-80	63	12.6	14 (10, 18)		24 (14, 30)		4 (2, 5)	
	81-90	16	3.2	15 (11, 28)		25 (13, 37)		4 (3, 5)	
Gender	Female	197	39.4	16 (11, 22)	0.045	15 (14, 33)	<0.001	4 (2, 6)	0.471
	Male	303	60.6	18 (11, 29)		31 (15, 38)		4 (2, 5)	
Marital status	Married	396	79.2	18 (11, 28)	0.008	31 (15, 37)	<0.001	4 (2, 5)	0.001
	Unmarried	30	6.0	18 (13, 21)		15 (13, 34)		5 (3, 6)	
	Divorced	17	3.4	16 (13, 17)		15 (15, 17)		2 (2, 3)	
	Widowed	57	11.4	12 (9, 19)		15 (13, 27)		3 (1, 4)	
Education Status	Illiterate	161	32.2	13 (8, 16)	<0.001	15 (14, 30)	<0.001	3 (2, 4)	<0.001
	Primary	20	4.0	16 (15, 18)		14 (13, 15)		4 (2, 5)	
	Middle	95	19.0	19(15, 29)		30 (15, 37)		4 (2, 5)	
	Secondary	110	22.0	16 (10, 25)		31 (15, 36)		4 (3, 6)	
	Degree	85	17.0	28 (20, 32)		37 (31, 40)		4 (3, 6)	
	Masters	29	5.8	29 (22, 31)		38 (33, 40)		5 (2, 5)	
Occupation of patient	Working	220	44.0	21 (14, 31)	<0.001	34 (28, 39)	<0.001	4 (2, 5)	0.007
	Unemployed	110	22.0	15 (9, 18)		15 (14, 30)		3 (2, 4)	
	House wife	106	21.2	15 (10, 18)		15 (14, 28)		3 (2, 5)	
	Retired	52	10.4	24 (10, 28)		30 (15, 37)		4 (2, 6)	
	Others	12	2.4	19 (18, 21)		35 (31, 37)		3 (1, 5)	
Health insurance	Yes	254	50.8	21 (15, 29)	<0.001	32 (15, 39)	<0.001	4 (3, 5)	<0.001
	No	246	49.2	14 (9, 18)		25 (14, 32)		3 (2, 4)	
Family history of the patient	Yes	204	40.8	19 (15, 29)	<0.001	31 (15, 38)	0.008	4 (3, 5)	0.004
	No	274	54.8	15 (10, 23)		29 (14, 34)		3 (2, 5)	
	Don’t know	22	4.4	17 (15, 19)		15 (14,38)		4 (4, 6)	
Patient history	Recently diagnosed	158	31.6	17 (12, 27)	0.847	32 (15, 39)	0.010	4 (2, 5)	0.120
	5 years	164	32.8	17 (12, 21)		22 (14, 37)		4 (2, 5)	
	10 years	83	16.6	17 (11, 28)		28 (15, 34)		4 (3, 6)	
	More than 10 years	95	19.0	17 (9, 30)		29 (14, 37)		3 (2, 5)	
How often do you attend your diabetes clinic	Every 3 months	183	36.6	22 (10, 32)	<0.001	32 (15, 39)	<0.001	4 (2, 6)	<0.001
	Every 6 months	167	33.4	16 (11, 22)		15 (14, 32)		3 (2, 4)	
	Once a year	82	16.4	16 (10, 20)		26 (14, 34)		4 (3, 5)	
	First time	68	13.6	17 (12, 19)		33 (16, 38)		4 (2, 5)	
Physical activity	Mild	198	39.6	16 (9, 21)	<0.001	26 (14, 34)	<0.001	3 (2, 5)	0.043
	Moderate	166	33.2	16 (12, 24)		29 (15, 35)		4 (2, 5)	
	High	136	27.2	21 (16, 30)		33 (19, 39)		4 (2, 5)	

**Table 2:** Summary of knowledge score of type II diabetespatients stratified by gender n = 500.

**Variables**		**Total**		**Female**		**Male**		***p*-value**
		**N**	**%**	**n**	**%**	**N**	**%**	
What is type II diabetes mellitus?	Is a raised blood sugar only	279	55.8	98	35.1	181	64.9	0.085
	Is a disease which affects any part of the body	61	12.2	24	39.3	37	60.7	
	I don't know	152	30.4	70	46.1	82	53.9	
What are the lifestyle risk factors for type II diabetes mellitus?	Eating too much	193	38.6	101	35.9	180	64.1	0.994
	Alcohol	269	53.8	45	26.5	125	73.5	0.003
	Eating too much fat and sugar	342	68.4	145	42.4	197	57.6	0.444
	Lack of exercise	281	56.2	90	33.5	179	66.5	0.073
	Smoking	170	34.0	76	39.4	117	60.6	<0.001
Identify treatment options for type II diabetes mellitus.	Dietary management	264	52.8	105	39.8	159	60.2	0.857
	Exercise	246	49.2	85	34.6	161	65.4	0.029
	Insulin therapy	164	32.8	61	37.2	103	62.8	0.481
	Regular BS monitoring	188	37.6	69	36.7	119	63.3	0.338
	Orally taken tablets	399	79.8	160	40.1	239	59.9	0.524
	Weight reduction	126	25.2	42	33.3	84	66.7	0.107
What are the symptoms of type II diabetes mellitus?	Excess thirst	339	67.8	129	38.1	210	61.9	0.371
	Loss of appetite	258	51.6	112	43.4	146	56.6	0.058
	Passing lots of urine	371	74.2	144	38.8	227	61.2	0.649
	Tiredness	351	70.2	135	38.5	216	61.5	0.51
	Weight loss	211	42.2	73	34.6	138	65.4	0.06
Complications of type II diabetes mellitus, if not treated	Burning sensation	256	51.2	108	42.2	148	57.8	0.191
	Eye problem or even blindness	168	33.6	49	29.2	119	70.8	0.001
	Amputation of limb	170	34.0	59	34.7	111	65.3	0.123
	Heart complications	348	69.6	139	39.9	209	60.1	0.707
	Kidney failure	192	38.4	57	29.7	135	70.3	<0.001
	Low blood sugar associated with dizziness, nausea and tremors	118	23.6	66	55.9	52	44.1	<0.001
	Nerve damage (neuropathy)	163	32.6	52	31.9	111	68.1	0.017
	Sexual dysfunction	192	38.4	17	26.2	48	73.8	0.019
	Stroke	123	24.6	47	38.2	76	61.8	0.765
Health risk factors for type II diabetes mellitus.	Family history	242	48.4	94	38.8	148	61.2	0.805
	Hyper-lipidemia	219	43.8	85	38.8	134	61.2	0.812
	Hypertension	261	52.2	95	36.4	166	63.6	0.151
	Obesity	267	53.4	98	36.7	169	63.3	0.187
Life style modification required to control diabetes	Dietary modification	309	61.8	125	40.5	184	59.5	0.54
	Exercise	197	39.4	68	34.5	129	65.5	0.072
	Weight reduction	235	47.0	74	32.6	153	67.4	0.005
	Nothing	46	9.2	19	41.3	27	58.7	0.781
Managing blood glucose levels is essential for reducing complications of type II diabetes mellitus?	Yes	354	70.8	144	40.7	210	59.3	0.365
	No	138	27.6	50	36.2	88	63.8	
HbA1c measures your average blood glucose level for the past.	Day	7	1.4	1	14.3	6	85.7	0.323
	Week	72	14.4	30	41.7	42	58.3	
	6-12 weeks	215	43.0	79	36.7	136	63.3	
	6 months	202	40.4	86	42.6	116	57.4	
The best method for glucose testing at home	Blood testing	296	59.2	111	37.5	185	62.5	0.444
	Urine testing	63	12.6	26	41.3	37	58.7	
	Both are equally good	137	27.4	60	43.8	77	56.2	
Infection is likely........glucose level	Lowers	76	15.2	30	39.5	46	60.5	0.045
	Raises	199	39.8	66	33.2	133	66.8	
	Has no effect	222	44.4	100	45.0	122	55.0	
Which one is the correct way to take care of feet is to:	Buy shoes a size larger than usual	66	13.2	50	75.8	16	24.2	<0.001
	Look at and wash them each day and keep them dry	246	49.2	76	30.9	170		
	Massage them with alcohol each day	54	10.8	17	31.5	37	68.5	
	Soak them for one hour each day	43	8.6	15	34.9	28	65.1	
	Don't know	91	18.2	39	42.9	52	57.1	
Numbness and tingling may be symptoms of:	Eye disease	37	7.4	12	32.4	25	67.6	<0.001
	Kidney disease	84	16.8	27	32.1	57	67.9	
	Liver disease	43	8.6	24	55.8	19	44.2	
	Nerve disease	160	32.0	43	26.9	117	73.1	
	Don't know	172	34.4	89	51.7	83	48.3	
Which of the following problem is usually not associated with diabetes mellitus?	Kidney	32	6.4	15	46.9	17	53.1	0.001
	Lung	136	27.2	42	30.9	94	69.1	
	Nerve	104	20.8	35	33.7	69	66.3	
	Vision	37	7.4	9	24.3	28	75.7	
	Don't know	188	37.6	94	50.0	94	50.0	

**Table 3:** Summary of attitude score of type II diabetes patients stratified by gender n = 500.

**Variables**		**Total**		**Female**		**Male**		***p*-value**
		**n**	**%**	**n**	**%**	**N**	**%**	
You should be regularly examined for having type II diabetes mellitus.	Strongly disagree	94	18.8	50	53.2	44	46.8	0.02
	Disagree	47	9.4	19	40.4	28	59.6	
	Neutral	45	9.0	17	37.8	28	62.2	
	Agree	201	40.2	77	38.3	124	61.7	
	Strongly agree	107	21.4	32	29.9	75	70.1	
Do you agree family members should be screened for type II diabetes mellitus?	Strongly disagree	141	28.2	77	54.6	64	45.4	<0.001
	Disagree	76	15.2	29	38.2	47	61.8	
	Neutral	100	20.0	35	35.0	65	65.0	
	Agree	95	19.0	27	28.4	68	71.6	
	Strongly agree	85	17.0	28	32.9	57	67.1	
Support from family and friends is important in dealing with type II diabetes mellitus.	Strongly disagree	164	32.8	84	51.2	80	48.8	0.001
	Disagree	41	8.2	20	48.8	21	51.2	
	Neutral	41	8.2	15	36.6	26	63.4	
	Agree	160	32.0	50	31.3	110	68.8	
	Strongly agree	90	18.0	27	30.0	63	70.0	
You need to cut down consumption of sweets.	Strongly disagree	164	32.8	85	51.8	79	48.2	0.001
	Disagree	31	6.2	11	35.5	20	64.5	
	Neutral	64	12.8	24	37.5	40	62.5	
	Agree	145	29.0	51	35.2	94	64.8	
	Strongly agree	91	18.2	23	25.3	68	74.7	
Type II diabetes mellitus is not seriously affects the marital relationship	Strongly disagree	98	19.6	25	25.5	73	74.5	<0.001
	Disagree	74	14.8	25	33.8	49	66.2	
	Neutral	39	7.8	11	28.2	28	71.8	
	Agree	97	19.4	39	40.2	58	59.8	
	Strongly agree	189	37.8	96	50.8	93	49.2	
Do you agree type II diabetes mellitus does not seriously affect daily activities?	Strongly disagree	64	12.8	18	28.1	46	71.9	0.005
	Disagree	99	19.8	31	31.3	68	68.7	
	Neutral	44	8.8	19	43.2	25	56.8	
	Agree	93	18.6	33	35.5	60	64.5	
	Strongly agree	193	38.6	95	49.2	98	50.8	
Do you agree physical activity can prevent risk of type II diabetes mellitus?	Strongly disagree	181	36.2	95	52.5	86	47.5	<0.001
	Disagree	35	7.0	20	57.1	15	42.9	
	Neutral	34	6.8	6	17.6	28	82.4	
	Agree	118	23.6	41	34.7	77	65.3	
	Strongly agree	127	25.4	33	26.0	94	74.0	
Do you agree maintaining a healthy weight is important for managing type II diabetes mellitus?	Strongly disagree	153	30.6	77	50.3	76	49.7	<0.001
	Disagree	31	6.2	16	51.6	15	48.4	
	Neutral	39	7.8	16	41.0	23	59.0	
	Agree	140	28.0	52	37.1	88	62.9	
	Strongly agree	130	26.0	33	25.4	97	74.6	
Do you agree complications of type II diabetes mellitus may be prevented if blood glucose level is well controlled?	Strongly disagree	114	22.8	54	47.8	59	52.2	0.071
	Disagree	22	4.4	11	47.8	11	52.2	
	Neutral	39	7.8	18	47.4	20	52.6	
	Agree	150	30.0	58	39.1	92	60.9	
	Strongly agree	165	33.0	53	32.1	112	67.9	

**Table 4:** Summary of practice score of type II diabetes patients stratified by gender n = 500.

**Variable**		**Total**		**Female**		**Male**		***p*-value**
		**n**	**%**	**n**	**%**	**n**	**%**	
Do you follow a dietary modification to control diabetes?	Yes- many times	191	38.2	76	39.8	115	60.2	0.477
	Yes- occasionally	243	48.6	100	41.2	143	58.8	
	No, never	64	12.8	21	32.8	43	67.2	
Have you tried to lose weight in the past to control diabetes?	Yes- many times	142	28.4	54	38.0	88	62.0	0.537
	Yes- occasionally	202	40.4	86	42.6	116	57.4	
	No, never	153	30.6	57	37.3	96	62.7	
Do you have a glucometer?	Yes	274	54.8	113	41.2	161	58.8	0.31
	No	223	44.6	82	36.8	141	63.2	
Do you check your blood sugar regularly?	Yes	235	47.0	97	41.3	138	58.7	0.427
	No	262	52.4	99	37.8	163	62.2	
Do you examine your foot regularly?	Yes	197	39.4	76	38.8	120	61.2	0.820
	No	298	59.6	119	39.8	179	60.2	

**Table 5:** Correlation between KAP scores and with demographic variables (Spearman’s rho).

**Variable**	**Knowledge**		**Attitude**		**Practice**	
	**Rho**	***P* value**	**Rho**	***P* value**	**Rho**	***P* value**
FSL (fasting sugar level)	-.113	.014	-.010	.829	-.109	.017
RBS (random blood sugar)	.128	.007	.080	.095	.069	.144
HbA1c (glycated haemoglobin)	-.013	.778	.132	.003	.014	.759
Age	-.170	<0.001	-.201	<0.001	.134	.003
Knowledge scores	-	-	.436	<0.001	.274	<0.001
Attitude scores	.436	<0.001	-	-	.191	<0.001
Practice scores	.274	<0.001	.191	<0.001	-	-

## Data Availability

The data and supportive information are available within the article.

## LIST OF ABBREVIATIONS

CI: Confidence Interval
DM II: Type II Diabetes Mellitus
FSL: Fasting Sugar Level
HbA1c: Glycatedhemoglobin
ICMR-INDIAB: Indian Council of Medical Research 2019-21
IEC: Institutional Ethics Committee
IQR: Interquartile Range
KAP: Knowledge, Attitude, and Practice
NFHS-5: National Family Health Survey 2019-21
QoL: Quality of Life
RBS: Random Blood Sugar
