# Supplementary material for: Gender-Based Disparities in Knowledge, Attitudes, and Practices Among Type-II Diabetes Patients in Punjab, India: Insights for Tailored Interventions
Source: Curr Pharm Des. 2025 Oct 24;32(21):1688–701. doi: 10.2174/0113816128409456251008231638 (PMC13478947; doi:10.2174/0113816128409456251008231638)
Supplement: Supplementary file 1 [file CPD-32-21-1688_SD1.pdf]

Supplementary Material

Gender-Based Disparities in Knowledge, Attitudes, and Practices Among Type-II Diabetes Patients in Punjab, India: Insights for Tailored Interventions

Sandeep Kaur[1,2], Sidharth Mehan[3,*] and Ghanshyam Das Gupta[1]

[1]Department of Pharmaceutics, ISF College of Pharmacy, Moga, Punjab, India; [2]Research Scholar, IK Gujral Punjab Technical University, Jalandhar, Punjab, India; [3]Department of Pharmacology, ISF College of Pharmacy, Moga, Punjab, India (Affiliated to IK Gujral Punjab Technical University, Jalandhar, Punjab, 144603, India)

1873-4286/XX $65.00+.00 **© XXXX Bentham Science Publishers**

Data collection form: Knowledge, Attitude and Practice Study in Type II Diabetes Mellitus Patients

Annexure-I

Informed consent form

Study Number ___________________

Full Name

____

E-mail address / contact no.

____

Age (years)

____

Hospital name:

____

I have no objection in participating in the study entitled '**Assessment and Evaluation of Knowledge, Attitude and Practice among patients suffering from Type II Diabetes mellitus'** carried by Ms. Sandeep Kaur.

I understand that my identity will not be disclosed to anyone without my permission and the data collected will be utilized only for research purpose. I promise to provide data sincerely to the best of my knowledge and co- operate to the follow up. I have the right to withdraw from the study any moment without giving any reason.

I confirm that I have read and understood the information sheet dated___________ for the above study.

Signature / thumb impression of the subject/ legally acceptable representative/ impartial witness: ______________________________

Signatory's name______________________ Date ________________________

Signature of the investigator ____________ Date ________________________

Annexure-II

Demographic profile

1. Weight distribution (kg).

- 41-50
- 51-60
- 61-70
- 71-80
- 81-90
- 91-100
- >101

2. Age distribution (years)

- 21-30
- 31-40
- 41-50
- 51-60
- 61-70
- 71-80
- 81-90
- >90

3. Gender

- Male
- Female

4. Marital status

- Married
- Unmarried
- Divorced
- Widowed

5. Level of education

- Illiterate
- Primary
- Middle
- Secondary
- Degree
- Masters
- PhD

6. Occupation

- Working
- Unemployed
- House wife
- Retired
- Others

7. Health insurance

- Yes
- No

8. Family history of diabetes

- Yes
- No
- Don't know

9. Patient history of type II diabetes mellitus.

- Recently diagnosed
- 5 years
- 10 years
- More than 10 years

10. Fasting sugar level (mg/dl)

____

11. Fasting sugar level: FSL

- <79-140 mg/dl
- 141-170 mg/dl
- 171-200 mg/dl
- 201-230 mg/dl
- 231-260 mg/dl
- 261-290 mg/dl
- >290 mg/dl

12. Random blood sugar (mg/dl)

____

13. Random blood sugar: RBS

- 201-230 mg/dl
- 231-260 mg/dl
- 261-290 mg/dl
- >290 mg/dl

14. Blood sugar: HbA1c (%)

____

15. Blood sugar: HbA1c

- Below 42 mmol/mol (6.0)
- 42 to 47 mmol/mol (6.0 to 6.4
- 48 mmol/mol (6.5 or ever)

16. How frequently do you visit your diabetic clinic?

- First time
- Every 3 months
- Every 6 months
- Once a year

17. Physical activity
- Mild
- Moderate
- High

Knowledge

Single-choice question*

K1. What is type II diabetes mellitus?
- Is a raised blood sugar only
- Is a disease which affects any part of the body
- I don't know

Please tick as many as apply*

K2. What are the lifestyle risk factors for type II diabetes mellitus?
- Alcohol………..yes/ no
- Eating too much ………..yes/ no
- Eating too much fat and sugar………..yes/ no
- Lack of exercise ………..yes/ no
- Smoking ………..yes/ no

K3. Identify treatment options for type II diabetes mellitus.
- Dietary management………..yes/ no
- Exercise………..yes/ no
- Insulin therapy ………..yes/ no
- Regular BS monitoring ………..yes/ no
- Tablets ………..yes/ no
- Weight reduction………..yes/ no

K4. What are the symptoms of type II diabetes mellitus?
- Excess thirst ………..yes/ no
- Loss of appetite………..yes/ no
- Passing lots of urine ………..yes/ no
- Tiredness………..yes/ no
- Weight loss ………..yes/ no

K5. Complications of type II diabetes mellitus, if not treated
- Burning sensation………..yes/ no
- Eye problem or even blindness ………..yes/ no
- Foot problems, potentially leading to amputation………..yes/ no
- Heart complications………..yes/ no
- Kidney failure ………..yes/ no
- Low blood sugar associated with dizziness, nausea and tremors………..yes/ no
- Nerve damage (neuropathy) ………..yes/ no
- Sexual dysfunction ………..yes/ no
- Stroke………..yes/ no

K6. Health risk factors for type II diabetes mellitus.
- Family history………..yes/ no

- Hyper-lipidemia ....yes/ no
- Hypertension ....yes/ no
- Obesity ....yes/ no
- Don't know

K7. Life style modification required to control diabetes:

- Dietary modification ....yes/ no
- Exercise ....yes/ no
- Weight reduction..........yes/ no
- Nothing

Single-choice question*

K8. Managing blood glucose levels is essential for reducing complication of type II diabetes mellitus?

- Yes
- No

K9. HbA1c measure your average blood glucose level for the past.

- Day
- Week
- 6–12 weeks
- 6 months

K10. The best method for glucose testing at home_____

- Blood testing
- Urine testing
- Both are equally good

K11. Infection is likely ________

- Lowers blood glucose level
- Raises blood glucose level
- Has no effect

K12. Which one is the correct way to take care of feet is to:

- Buy shoes a size larger than usual
- Look at and wash them each day and keep them dry
- Massage them with alcohol daily
- Soak them for one hour daily
- Don't Know

K13. Numbness and tingling may be symptoms of:

- Eye disease
- Kidney disease
- Liver disease
- Nerve disease
- Don't Know

K14. Which of the following problem is usually not associated with diabetes mellitus?

- Kidney

- Lung
- Nerve
- Vision
- Don't Know

Attitude

*Single-choice question**

A1. You should be regularly examined for having type II diabetes mellitus.
- Strongly disagree
- Disagree
- Neutral
- Agree
- Strongly agree

A2. Do you agree family members should be screened for type II diabetes mellitus?
- Strongly disagree
- Disagree
- Neutral
- Agree
- Strongly agree

A3. Support from friends and family is important in dealing with type II diabetes mellitus.
- Strongly disagree
- Disagree
- Neutral
- Agree
- Strongly agree

A4. You need to cut down consumption of sweets:
- Strongly disagree
- Disagree
- Neutral
- Agree
- Strongly agree

A5. Type II diabetes mellitus is not seriously affects the marital relationship:
- Strongly disagree
- Disagree
- Neutral
- Agree
- Strongly agree

A6. Do you agree type II diabetes mellitus does not seriously affects daily activities?
- Strongly disagree
- Disagree
- Neutral
- Agree
- Strongly agree

A7. Do you agree physical activity can prevent risk of type II diabetes mellitus?

- Strongly disagree
- Disagree
- Neutral
- Agree
- Strongly agree

A8. Do you agree maintaining a healthy weight is important for managing type II diabetes mellitus?

- Strongly disagree
- Disagree
- Neutral
- Agree
- Strongly agree

A9. Do you agree complications of type II diabetes mellitus may be prevented if blood glucose level is well controlled?

- Strongly disagree
- Disagree
- Neutral
- Agree
- Strongly agree

Practice

*Single-choice question**

P1. Do you follow a dietary modification to control diabetes?

- Yes- Many times
- Yes- occasionally
- No, never

P2. Have you tried to lose weight in the past to control diabetes?

- Yes- Many times
- Yes- occasionally
- No, never

P3. Do you have a glucometer?

- Yes
- No

P4. Do you check your blood sugar regularly?

- Yes
- No

P5. Do you examine your foot regularly?

- Yes
- No
